# Prevalence of Psychiatric Symptoms in ALL Patients during Maintenance Therapy

**Published:** 2015-04-20

**Authors:** H Farhangi, Z Badiei, F Moharreri

**Affiliations:** 1 Assistant Professor of Pediatric Hematology & Oncology, faculty of medicine, Mashhad University of Medical Sciences, Mashhad, Iran.; 2 Associated Professor of Pediatric Hematology & Oncology, faculty of medicine, Mashhad University of Medical Sciences, Mashhad, Iran.; 3 Psychiatry and Behavioral Sciences Research Center, Ibn-e-Sina Hospital, Faculty of Medicine, Mashhad University of Medical Sciences, Mashhad, Iran.

**Keywords:** Acute Lymphoblastic Leukemia, Adolescents, Anxiety, Behavioral Disorders, Children, Depression

## Abstract

**Background:**

Cancer diagnosis may cause deep emotional and affective problems in patients and their families. Nowadays, however, despite its rising prevalence, cancer is no longer synonymous with death. Given the significance of emotional well-being in cancer patients, we decided to assess the frequency of psychological problems in seven to seventeen year-olds with acute lymphoblastic leukemia.

**Materials and Methods:**

Our sample included 42 children and adolescents with ALL referred to pediatric hematology department of Dr. Sheikh hospital, who were put under maintenance course of the treatment. Psychiatric disorders such as anxiety, depression and behavioral disturbances were examined by using RCMAS, CDI and SDQ questionnaires respectively.

**Results:**

The entire population showed depressive symptoms.59.5% of patients (25 person) suffered from anxiety and 26.2% (11 person) had behavioral problems. No significant relation was found between depressive symptoms, and age (p=0.77), sex (p=0.97), length of disease (p=0.50), and type of treatment (p=0.064). Anxiety did not show any significant relation with age (p= 0.63), sex (p= 0.32), length of disease (p= 0.16) and treatment type (p= 0.064).Similarly behavioral disturbances did not indicate any suggestive relation with age (p= 0.20), sex (p= 0.56), length of disease (p= 0.81) and type of treatment (p= 0.19).

**Conclusion:**

Our findings suggest a high prevalence of psychiatric disorders in children and adolescents with ALL. It is strongly recommended, therefore, that besides somatic symptoms, careful attention be paid to psychological disorders. This can prevent rapid development of the disease reduce treatment costs, and improve the quality of life for both patients and their families.

## Introduction

Nowadays, cancer, though a serious and chronic disease, is not incurable (1-4). Emergence of disease symptoms such as weakness, fatigue, pain, and loss of abilities makes them feel closer to death, and gradually causes depression in most patients. Despair, disappointment, anger, and at times suicidal thoughts are among the most frequently reported feelings (3). Recent medical improvements in the field have extended life expectancy in patients suffering from cancer. Given the adverse effects of depression and stress, it is important to pay special attention to psychological and psychosocial aspects of the disease and to take appropriate consulting measures in order to improve the quality of patients’ lives. Childhood cancer mortality rate in the US is about 8300 cases per year (3). Leukemia and brain tumors are the most common malignancies in children (3). 

Lymphoblastic leukemia is a disorder in maturation and differentiation of lymphoid precursors, which causes them to proliferate uncontrollably (3).Acute lymphoblastic leukemia is the most frequent malignancy during childhood and in children under the age of 15 (3). Depression and anxiety are two common psychiatric disorders in patients suffering from cancer (1). Of the two, depression is more important, as it affects functional status and quality of life (life quality) more harmfully (1).As to anxiety, the most common type with cancer patients is generalized anxiety disorder (1). Since the 1960s, depression has been known as a mental comorbidity affecting about 10 to 25 percent of cancer patients (1, 5). Prevalence rates vary according to psychiatrists' different conceptualizations of depression and its defining criteria, measurements methods, and populations studied (5). Given the high prevalence of cancer and leukemia in children and adolescents, psychotherapeutic techniques are more seriously regarded as an appropriate means to significantly prevent complications and mental problems in the course of treatment. The objective of the present paper is to provide data on psychiatric symptoms and report effective therapeutic methods in patients with ALL.

## Methods

This is a descriptive cross-sectional study. The sample was selected from among ALL cases referred to pediatric hematology department of Dr. Sheikh hospital, from November 2011 to May 2012. All patients who fitted the criteria entered the study. Our criteria were the following: age 7 to 17; diagnosed with ALL; having granted informed consent; being under maintenance course of treatment. Exclusion criteria included: unwillingness to take part in the study; being bereft of the care of either or both parents; leukemia involvement of the central nervous system. Finally the sample included 42 Patients. Physicians provided the patients and their families with explanations on the study and having received their informed consent, gave them three questionnaires. We used standard questionnaires with confirmed validity and reliability to measure anxiety, depression and behavioral disorders. The collected data were then analyzed by SPSS 11.5 and chi-square test. 

**Sampling instruments**

Anxiety, depression and behavioral disorders were measured using RCMAS test, CDI test, and SDQ test respectively. 

**Revised Children’s Manifest Anxiety Scale (RCMAS** **)**

Designed by Reynolds & Richmond in 1987, RCMAS questionnaire is used to assess anxiety level in children and adolescents (6). It comprises of 37 questions to which the patient responds by either “yes” or “no” according to his/her personal feelings and thoughts. Based on patient’s answers, the value of each item is either zero or one. At the end, patient’s general score is calculated. If his/her general score is between 0 to 9, the patient is in a normal range of anxiety. Score 10 and above indicates an abnormal anxiety level. Patients whose anxiety is in the abnormal range are further classified into 3 categories: mild, moderate and severe. Those who score 10 to 15 are classified as mildly anxious. Scores 16 to 30 indicate moderate anxiety and scores above 30 suggest severe levels

**Children Depression Inventory (CDI)**

This questionnaire, designed by Kovacs& Beckin 1981, is used to evaluate behavioral, cognitive and emotional symptoms in 7 to 17 year old children and adolescents. A wide range of depression symptoms such as anxious mood, hedonic capacity, hopelessness, low self-evaluation and interpersonal problems can be measured by CDI test (7). Reliability coefficients of this questionnaire have been reported 0.8 and higher (7). It is a self-report questionnaire consisting of 27 questions on patient’s activities, feelings and choose between three options, a, b or c. Based on his/her answer a score of 0 to 2 is calculated for each question. Choosing option a, assigns no score to the item (0); Choosing options b and c assigns respectively 1 and two score to the item. Patients who score 10 are in the abnormal level and are classified in three levels accordingly: those who score 10 to 15 are categorized as mildly depressed; scores 16 to 25 suggest moderate depression, and scores above 40 indicate a severe level of depression. 

**Strengths and Difficulties Questionnaire (SDQ)**

This questionnaire is used to evaluate behavioral problems in 3 to about 16 year olds. The original English version was created by Robert Goodman at the Institute of Psychiatry in London in 1997 (8). It consists of 25 sentences on which patients are asked to provide a comment according to their mental states in the past six months. Each sentence has three options (i.e. “it is not correct”, “it is a little correct”, and “it is correct certainly”). Parents must choose one of the options and mark it. Of the 25 questions, each 5 question set constitutes one criterion. These five criteria are emotional symptoms criterion, behavioral problems criterion, hyperactive criterion, problems with peers criterion and being social criterion. Score 1 is assigned to the option of “it is a little correct”. For the two other options, calculation is different and the score can vary between 0 to 10 according to the question concerned. Score is calculated for each criterion, which may range from 0 to 10. Then, general criteria is calculated by adding up all criteria except “being social”. General criteria ranges from 0 to 40. Results can be classified under normal, marginal and abnormal (Table II).

## Results

The mean age of patients was 9.83±2.89. Other demographic data shown in TableI. Also, frequency and severity of anxiety and depression are seen in diagram 1 and 2. For behavioral problems, 23, 8 and 11 patients were in normal, marginal and abnormal group respectively. For different domains, frequency of these problems is shown in Table II. Severity of anxiety and depression and also behavioral difficulties didn’t have any relation with age and sex in these patients. Our study shows that although level of anxiety and depression are decreased with increasing length of treatment and on the contrary behavioral difficulties are expanded, there isn’t any relation between duration of treatment and severity of depression, anxiety and behavioral problems. The study shows that anxiety and behavioral problems have more severity in high risk patients than ones with standard risk. However, this difference wasn’t significant statistically. 

**Table I:** Demographic and Disease Characteristics

	n	%
Number of patients	42	
Gender		
Male Female	29 13	69 31
Age (years)		
7-10 11-17	35 17	83.4 16.7
Treatment		
Standard risk High risk	30 12	71.4 26.8
Time since diagnosis		
<6 month 6-12 month >12 month	3 2 37	7.1 4.8 88.1

**Table II:** Behavioral difficulties in children with ALL during maintenance treatment

Behavioral difficulties	Normal (score)	Marginal (score)	Abnormal (score)
General score	54.8% (0-13)	19% (14-16)	26.2% (17-40)
Emotional symptom criteria	57.1% (0-3)	11.9% (4)	31% (5-10)
Behavioral problems criteria	50% (0-2)	16.7% (3)	33.3% (4-10)
Hyperactive criteria	73.8% (0-5)	16.7% (6)	9.5% (7-10)
Criteria of problems with peers	45.2% (0-2)	23.8% (3)	31% (4-10)
Being social criteria	88.1% (6-10)	7.1% (5)	4.8% (0-4)

**Figure 1:**
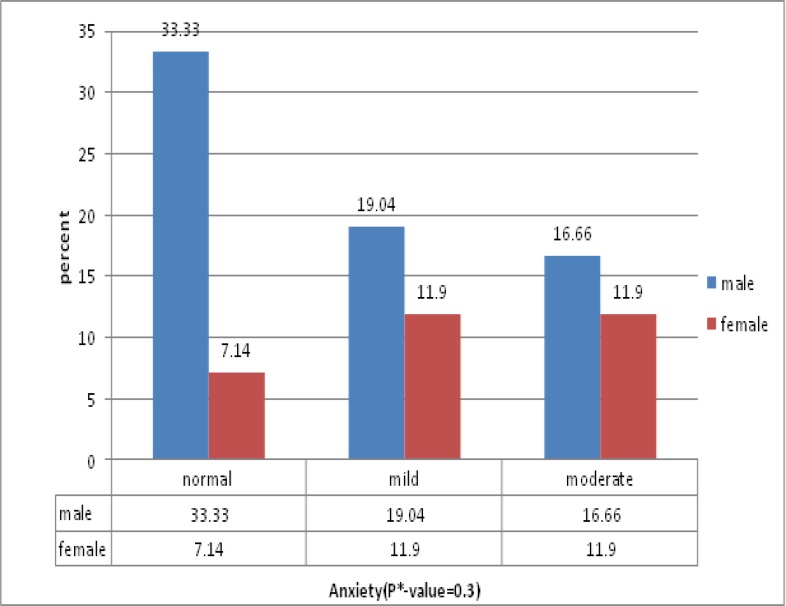
Anxiety based on gender and percent of patients. *P- value less than 0.05 was considered as significant

**Figure 2:**
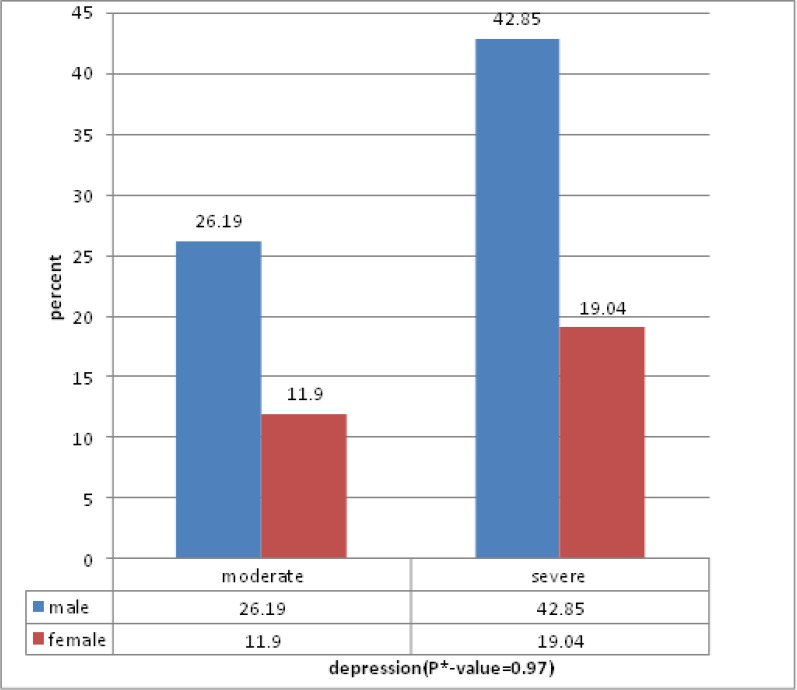
Depression based on gender and percent of patients. *P- value less than 0.05 was considered as significant

## Discussion

The mean age of patients was 9.83±2.896. Our findings show that 59.5% of patients have anxiety, whereas depression is seen in all patients approximately. 

Although there are a few studies about depression in children and adolescents with cancer, more investigative groups conducted significant studies in this field and reported that children with cancer aren’t more depressed than healthy ones(8). Allen et al. performed a study on 42 adolescents with different kinds of cancer, aged 12 to 20 years old, and found that these patients were not more depressed or anxious in comparison with control group (8). In general population, girls have more anxiety than boys. In our study, mild and moderate anxiety levels belongs to female gender. However, there is no significant relation between level of anxiety and sex variable based on chi-square test. According to our findings, adolescent patients are more anxious than child patients. This is inconsistent with the trend in public population, wherein anxiety symptoms decrease with the advance ofage (9). On the other hand, our findings show that prevalence of moderate anxiety is more in adolescents while in general population, anxiety symptoms are decreased with increasing age. However, there is no significant relation between level of anxiety and age according to chi-square test. In a study conducted by Hinz et al. in 2009 in Germany, 1529 patients with cancer were studied. Findings suggest psychological symptoms such as anxiety and depression are age-related. In early ages they are more prevalent but gradually decrease with age (9).Our findings, however, showed that anxiety increases with age. Level of anxiety decreased with length of disorder although there was no significant relation between them based on chi-square test. No study was found about this relation. Severe depression is more in male gender while in females moderate depression is more. Also, before puberty, prevalence of depression is equal in boys and girls, however, girls are more prone to depression after it(7). Moderate depression and severe depression are higher in adolescents and children respectively. Generally, depression in adolescents is more than children while severe depression is higher in children in our study. Matzo et al performed a study on 80 children and adolescents with cancer in 2008 in Greece. Like our study, the mean score of depression is more in boys but this difference was not statically significant (4). Contrary to our study, the mean score of depression is higher in female gender in a study conducted by Hinz et al (9). Also, the mean score of depression was more in cancer adolescent patients while our results were different (9). Our findings showed that with the increase of length of disorder, prevalence of moderate depression also increases.

However, our findings do not support severe depression. But in a study performed by Esmaeeli et al in Mashhad in 2013, there was a significant correlation between duration of illness and severity of depression (10). In patients who received high risk treatment, moderate depression was common. Severe depression was more common in patients who received standard risk treatment. However there was no significant relation between level of depression and type of treatment. In a study by Firoozi, depressed boys were shown to have more behavioral disturbances. Our study also suggests behavioral disorders to be commoner with boys and therefore supports Firoozi’s findings (2). Emotional symptoms, however, are more common with girls. Likewise, behavioral distresses are more in children age group, while Dolgin et al show different results and conclude that adolescents show more severe behavioral disorders (11). Abnormal emotional symptoms are slightly more in males (compared to females) and are higher in children. Hyperactive criteria are generally higher in males and adolescents. Also, males and children show higher criteria for problems with peers. Moreover, abnormal scores in being social criterion is more frequent in females and in children.

## Conclusion

Finally, based on findings, all patients showed depression symptoms. Also, 59.5% and 26.2% of patients had anxiety and behavioral problems, respectively. Moreover, we found that psychological difficulties did not have any significant relation with age, sex, length of disorder, complication and type of treatment. Therefore, in addition to somatic symptoms, physicians must also consider the treatment of psychiatric difficulties in patients with acute lymphoblastic leukemia for in-time prevention, improving quality of life, and minimizing treatment costs. Some of the limitations of this study include a relatively small sample size, lack of ideal conditions for conducting psychiatric interviews with patients, not taking patients and their families’ financial and socio-cultural status into account in our assessments, and dispensing with patient’s self-awareness of their disease as probably influential factors. In fact, all these factors affect anxiety and depression levels and are, therefore, worthy of further analysis.

## Conflict of interest

The authors have no conflict of interest.
